# SUPPORTIVE CARE NEEDS AND INTERVENTIONS IN PANCREATIC CANCER PATIENTS AND THEIR CAREGIVERS: A SCOPING REVIEW

**DOI:** 10.1093/geroni/igae098.4324

**Published:** 2024-12-31

**Authors:** Liang Fu, Su Hyun Kim, Matt Hayward, Candice Vieira, Alexander Parikh, Lixin Song

**Affiliations:** University of Texas Health Science Center at San Antonio, San Antonio, Texas, United States; Kyungpook National University, Daegu, Republic of Korea; Kyungpook National University, Daegu, Republic of Korea; Kyungpook National University, Daegu, Republic of Korea; Kyungpook National University, Daegu, Republic of Korea; University of Texas Health Science Center at San Antonio, San Antonio, Texas, United States

## Abstract

Pancreatic cancer remains one of the most lethal cancers and predominantly affects older adults. This scoping review aims to provide a comprehensive review of the literature regarding supportive care needs and corresponding interventions in patients with pancreatic cancer and their family caregivers. We conducted this scoping review following the Joanna Briggs Institute Manual for Evidence Synthesis. We searched five English databases using ‘pancreatic cancer,’ ‘patients/caregivers,’ ‘supportive care,’ and ‘needs’ in January 2024. We summarized the data employing the Supportive Care Framework. Of the 4,342 references identified, 41 were included. Among the 31 descriptive studies, the top four highest supportive care needs were informational (n = 24), physical (n = 10), emotional (n = 9), and practical needs (n = 8). In the 10 experimental studies, most of the interventions in involved pain/symptom management (n = 8), following by adjustment/supportive counselling (n = 6), psycho-educational service or activity (n = 4), and orientation/ongoing patient and family education (n = 4). Four studies demonstrated statistically significant improvements in outcomes for intervention groups compared to control groups. Patients with pancreatic cancer and their family caregivers experienced a spectrum of supportive care needs, particularly informational needs. Several intervention strategies were applied to address these supportive care needs, but only a few demonstrated statistically significant improvements in outcomes. These findings enhance our understanding of the supportive care needs and appropriate interventions for patients with pancreatic cancer and their family caregivers, providing a foundation for further research to address these needs through a health equity lens.

